# Emergency Management of Ventilation Failure Through Blocked Tracheostomy Tube in a Paediatric Patient

**DOI:** 10.7759/cureus.26873

**Published:** 2022-07-14

**Authors:** Ahmed Bilal Akhtar, Ahsun Khan, Huma Saleem, Zahra Mannan, Muhammad Naveed Azhar

**Affiliations:** 1 Anesthesia and Critical Care, Shaukat Khanum Memorial Cancer Hospital and Research Centre, Lahore, PAK; 2 Anesthesia and Critical Care, Shaukat Khanum Memorial Cancer Hospital and Research Centre, lahore, PAK

**Keywords:** management, emergency, ventilation, blockage, airway, anaesthesia, paediatrics, tracheostomy

## Abstract

The most common complication of tracheostomy tubes in children is blockage of the tube. We report a case where ventilation after induction of anaesthesia was not possible even though there were no signs of impending obstruction. An eight-year-old child, recently diagnosed with left tonsillar embryonal rhabdomyosarcoma, presented for an MRI face and bone marrow biopsy before starting treatment. Due to difficulty in breathing, the patient had undergone a tracheostomy at a different institute and a size six uncuffed tracheostomy tube was in situ. There was difficulty in ventilating the patient due to blockage in the tracheostomy tube which was addressed and the patient was discharged after successful completion of both the procedures. This case highlights the importance of following an emergency algorithm for failure to ventilate in a patient with a tracheostomy tube, identifying the cause and treating it.

## Introduction

Tracheotomy means making a surgical opening in the trachea and tracheostomy denotes the formation of the orifice in the skin which leads to the trachea [1]. The indications of tracheostomy include upper airway obstruction, prolonged mechanical ventilation, bronchial toilet, part of a surgical procedure (laryngectomy) and airway protection in diseases like bulbar palsy [2]. Tracheostomy tubes can be of various sizes, cuffed or uncuffed and may be fenestrated. The different sizes of the tube are as per the inner diameter, outer diameter, length, and curvature of the tube [3].

The complications of a tracheostomy can be divided into three categories. Early complications include haemorrhage, aspiration, pneumothorax and failure of the procedure. Short-term problems can be a blockage, tube displacement, pneumothorax, surgical emphysema, infection, delayed haemorrhage, tracheal necrosis and trachea-arterial fistula. Lastly, long-term complications such as tracheomalacia, tracheal stenosis, tracheocutaneous fistula and decannulation problems can occur [4]. It is important for all the physicians who are dealing with airway to have knowledge regarding the tracheostomy tube.

This article was previously presented as a poster at 20th Shaukat Khanum Cancer Symposium on November 7, 2021.

## Case presentation

An eight-year-old boy presented to the ENT specialist with a history of a sore throat for one month. On physical examination, a mass was noted on the left side of the base of the tongue. CT advised by the specialist showed a left tonsillar soft tissue density lesion with no evident necrosis to suggest abscess formation. After the tissue biopsy patient developed dysphagia and complained of shortness of breath. A tracheostomy was done to relieve the symptoms and a nasogastric tube was passed for feeding.

The child came to us for baseline magnetic resonance imaging (MRI) face and bone marrow biopsy under General Anaesthesia before commencing chemotherapy. Apparently, the child was sitting comfortably with a tracheostomy tube in situ, with no visible signs and symptoms of distress or shortness of breath. Baseline vitals were within normal range. The stoma site was normal on examination as well as the airflow which was checked by placing a hand near the opening of the tracheostomy tube. On examination, the tongue was completely abutting the oral cavity.

As per the initial plan, inhalational induction was started by connecting the circuit to the tracheostomy tube. On connecting, the circuit child became anxious and wanted to be in a sitting position. The circuit was disconnected and reattached in a sitting position with the tracheostomy tube. Sevoflurane was started at 4% initially with visible chest rise, movement of the rebreathing bag, end-tidal carbon dioxide, respiratory rate of 20-24 and saturation of 99%.

After sedating the patient, he was placed in the supine position. Subsequently, the end-tidal carbon dioxide was absent with no movement of the rebreathing bag but with visible exertion of the patient to breathe and drop in saturation. A fast sweep was done starting from patient end to machine, checking all possibilities for a blockage in parts like circuit, expiratory and inspiratory valve, adjustable pressure-limiting (APL) valve, soda lime, heat-moisture exchange (HME) filter and vaporizer. On auscultation, harsh breathing sounds were audible in both lungs.

A call for help was made once the cause could not be identified. Upon disconnecting the circuit from the tracheostomy tube patient was breathing comfortably in room air and maintaining saturation. The machine was checked manually but there was no fault. The suctioning catheter could not be passed through the tracheostomy tube. The probable cause seemed to be blockage of the tracheostomy tube.

After the arrival of help, paediatric bougie was pushed through the tube. Following an initial resistance, there was a sudden passage of bougie through a tracheostomy tube. After removing the bougie, the circuit was connected to a tracheostomy tube which showed high end-tidal carbon dioxide (50-60 mmHg). But the end-tidal carbon dioxide vanished again with the patient breathing irregularly. It was more evident by now that there was a blockage in the tracheostomy tube, stoma or trachea. Surgery team was called in and it was collectively decided to move the patient to Operation Theatre (controlled environment) for doing fiber-optic bronchoscopy before tracheostomy tube replacement.

**Figure 1:**
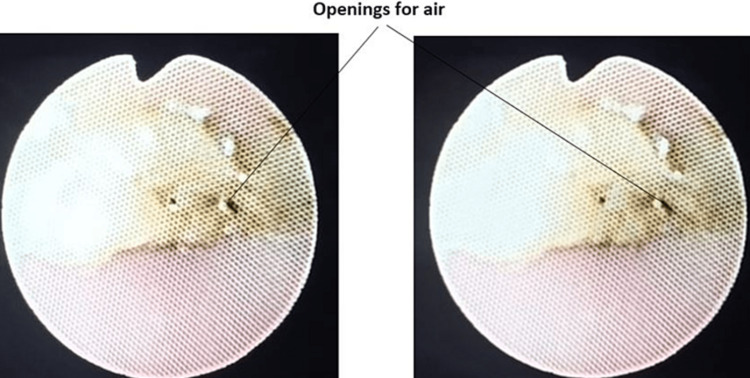
Lumen of tracheostomy tube obstructed with crusting.

On doing the fiber-optic bronchoscopy, Figure 1 shows the visible blockage due to crusted mucus plug. Subsequently big size Nelaton catheter was used to break the crusted secretions. Ventilation of the patient became smoother. Single cannula Tracheostomy tube was replaced with a double cannula cuffed tracheostomy tube and secured properly. After successfully changing the tube, patient underwent both the procedures and was discharged.

## Discussion

As per the Royal College of Anaesthetists fourth National Audit Project, the most common tracheostomy-related critical incidents were due to displacement [5]. The most common causes of death in children due to tracheostomy are tube obstruction, misplacement, and accidental decannulation [6]. As per the literature, there are a few more documented cases of obstruction in tracheostomy tubes but the causes are different such as fragmented tube [7] or a defective pilot [8].

In this case, the patient had a blocked tracheostomy tube but with no visible signs and symptoms of respiratory distress. The narrow opening through the crusting had allowed the patient to breathe under negative pressure. Once attached to the circuit the increase in resistance caused increased work of breathing and hence the difficulty in breathing. As the circuit would be detached, the patient was able to breathe in through the environment with less resistance. The circumstances made it difficult to diagnose the obstruction and it could easily be missed.

Patients with an existing tracheostomy can present for surgery. It is essential to check the type, size, distance of tube end from carina, presence of inner cannula and patency of tracheostomy tube in such patients [9,10]. Speaking valves can be used incorrectly and these along with the small humidifying devices can easily become blocked with secretions [11]. The upper end of the airway needs to be assessed for emergency intubation and a backup ventilation strategy should be devised.

The important points in the emergency management of tracheostomy tube as per the multidisciplinary guidelines for the management of tracheostomy and laryngectomy include calling for help, look, listen and feel for breathing at the mouth and tracheostomy site. Use of capnograph. Check pulse if not palpable, start Cardiopulmonary resuscitation (Chest compressions) and call the resuscitation team. If there is a pulse then oxygenation of the patient should be prioritized. Suction is only attempted after removing a potentially blocked inner tube. Oxygen is applied to both potential airways. A blocked or displaced tracheostomy tube is removed as soon as this is established and not as a last resort [11].

## Conclusions

Paediatric patients have lower respiratory reserves. Any reason causing respiratory compromise can lead to fatal consequences. Especially when the airway is difficult and there is no other way of resuscitating the patient. Our case represents the successful management of an emergency situation following basic guidelines of tracheostomy troubleshooting. Always follow the basics which is to oxygenate the patient and look for the cause.
